# Outbreaks of Neuroinvasive Astrovirus Associated with Encephalomyelitis, Weakness, and Paralysis among Weaned Pigs, Hungary

**DOI:** 10.3201/eid2312.170804

**Published:** 2017-12

**Authors:** Ákos Boros, Mihály Albert, Péter Pankovics, Hunor Bíró, Patricia A. Pesavento, Tung Gia Phan, Eric Delwart, Gábor Reuter

**Affiliations:** ÁNTSZ Regional Institute of State Public Health Service, Pécs, Hungary (A. Boros, P. Pankovics, G. Reuter);; University of Pécs, Pécs (A. Boros, P. Pankovics, G. Reuter);; Ceva Phylaxia Ltd., Budapest, Hungary (M. Albert);; SHP Ltd., Kaposvár, Hungary (H. Bíró);; University of California, Davis, California, USA (P.A. Pesavento);; Blood Systems Research Institute, San Francisco, California, USA (T.G. Phan, E. Delwart);; University of California, San Francisco (E. Delwart)

**Keywords:** viruses, encephalomyelitis, domestic pig, swine, epidemic, neurovirulent, paralysis, astrovirus, paraplegia, outbreak, Hungary, meningitis/encephalitis, respiratory infections

## Abstract

A large, highly prolific swine farm in Hungary had a 2-year history of neurologic disease among newly weaned (25- to 35-day-old) pigs, with clinical signs of posterior paraplegia and a high mortality rate. Affected pigs that were necropsied had encephalomyelitis and neural necrosis. Porcine astrovirus type 3 was identified by reverse transcription PCR and in situ hybridization in brain and spinal cord samples in 6 animals from this farm. Among tissues tested by quantitative RT-PCR, the highest viral loads were detected in brain stem and spinal cord. Similar porcine astrovirus type 3 was also detected in archived brain and spinal cord samples from another 2 geographically distant farms. Viral RNA was predominantly restricted to neurons, particularly in the brain stem, cerebellum (Purkinje cells), and cervical spinal cord. Astrovirus was generally undetectable in feces but present in respiratory samples, indicating a possible respiratory infection. Astrovirus could cause common, neuroinvasive epidemic disease.

Astroviruses are small, nonenveloped viruses with single-stranded 6.2–7.8 kb RNA genome of positive polarity (*1**,**2*). The family *Astroviridae* is currently divided into 2 genera: the genus *Mamastrovirus* of mammal-infecting viruses and the genus *Avastrovirus* of avian viruses (*3**,**4*). The genetically heterogenic astroviruses that are widespread among mammals and birds are generally associated with gastroenteritis, less commonly with respiratory disease, and rarely encephalitis or disseminated infections (*2**,**5**–**19*). Astrovirus infections with central nervous system (CNS) involvement were reported recently in mink, human, bovine, ovine, and swine hosts (the latter in certain cases of AII type congenital tremors) (*5**,**6**,**12**–**14*). Most neuroinvasive astroviruses belong to the Virginia/Human-Mink-Ovine (VA/HMO) phylogenetic clade and cluster with enteric astroviruses identified from asymptomatic or diarrheic humans and animals (*15**,**16*). Recent research shows that pigs harbor one of the highest astrovirus diversities among mammals examined (*3**,**15**,**20*). Porcine astroviruses (PoAstVs) were identified mainly from diarrheic fecal specimens, less commonly from respiratory specimens, although the etiologic role of astrovirus infection in gastroenteritis or in other diseases among swine is not settled (*3**,**9**,**20**–**23*). We report the detection of neuroinvasive porcine astrovirus type 3 (Ni-PoAstV-3) by reverse transcription PCR (RT-PCR) and in situ hybridization (ISH) in recent and archived CNS samples of newly weaned paraplegic pigs from 3 highly prolific swine farms in Hungary.

## Materials and Methods

### Sample Collection and Handling

During November 2015–July 2017, we collected multiple tissue samples from 5 paraplegic and 5 asymptomatic pigs at the index farm located in Hungary (GD; specific location redacted) (Table 1). We also tested nasal and anal swab pairs collected by using polyester-tipped swabs from another 5 paraplegic and 13 healthy animals. We washed tissue samples twice in 10 mmol/L phosphate buffered saline (PBS) to remove excess blood and held them at −80°C until total RNA extraction. For formalin-fixed, paraffin-embedded (FFPE) blocks, we fixed the dissected samples (Table 2) with buffered 8% formaldehyde, dehydrated and embedded into paraffin.

**Table 1:** Data on 5 symptomatic and 5 control newly weaned pigs from a farm in Hungary and results of PoAstV-3 screening by nested RT-PCR of samples collected during 2015–2017*

Data	Symptomatic animals†						Asymptomatic control animals†				
	GD-1	GD-2	GD-3	GD-4	GD-5		GD-6	GD-7	GD-8	GD-9	GD-10
Collection month	2016 Mar	2016 Mar	2016 Jul	2016 Jul	2015 Nov		2016 Jul	2017 Jun	2017 Jun	2017 Jun	2017 Jun
Age, d	25	25	25	25	35		35	25	25	25	35
Clinical signs (disease stage)	PP (1)	PP (1)	PP (3)	PP (3)	PP (3)		None	None	None	None	None
Brain stem	+	+	+	+	+		– (–)	– (–)	– (–)	– (–)	– (–)
Spinal cord											
Cervical	NA	NA	+	+	NA		NA	– (–)	– (–)	NA	NA
Thoracic	NA	NA	+	+	NA		NA	– (–)	– (–)	NA	NA
Lumbar	+	NA	+	+	+		– (–)	– (–)	– (–)	NA	NA
Nasal mucosa	– (+)‡	+‡	+	+	NA		– (–)	– (–)‡	– (–)‡	– (–)‡	– (–)‡
Lung	NA	NA	+	+	NA		NA	NA	NA	NA	NA
Tonsils	NA	– (–)	+	+	+		– (–)	NA	NA	NA	NA
Salivary glands	NA	NA	– (+)	+	NA		NA	NA	NA	NA	NA
Myocardium	NA	+	NA	NA	+		NA	NA	NA	NA	NA
Feces	– (–)	NA	– (+)	– (+)	NA		NA	– (–)	– (–)	– (–)	– (–)
Ileum	NA	NA	– (–)	– (+)	– (–)		– (–)	NA	NA	NA	NA
Lymph nodes											
Mesenterial	NA	– (–)	– (–)	– (–)	NA		– (–)	NA	NA	NA	NA
Submandibular	NA	NA	– (+)	+	NA		NA	NA	NA	NA	NA
Urine	NA	NA	– (–)	– (–)	NA		NA	NA	NA	NA	NA
Kidney	NA	NA	– (–)	– (–)	NA		NA	NA	NA	NA	NA
Liver	NA	NA	– (+)	– (+)	NA		NA	NA	NA	NA	NA
Spleen	NA	NA	– (–)	– (–)	NA		NA	NA	NA	NA	NA
Serum	NA	NA	+	+	NA		NA	NA	NA	NA	NA

*We collected tissues from 5 affected pigs with encephalomyelitis and PP and 5 asymptomatic control animals from the index farm. The screening nested RT-PCR primers are designed to the RNA-dependent RNA polymerase region of PoAstV-3. NA, no available sample; PoAstV-3, porcine astrovirus type 3; PP, posterior paraplegia; RT-PCR, reverse transcription PCR; +, positive; −, negative. †Symbols indicate results from first PCR reactions; symbols in parentheses indicate results from second (nested) RT-PCR reactions. ‡Nasal swab sample.

**Table 2:** Results of PoAstV-3 detection, histology, and ISH analyses using formalin-fixed, paraffin-embedded blocks of samples from 3 symptomatic newly weaned pigs from a farm in Hungary and samples from 2 other farms with symptomatic pigs*

Farm ID	Collection year	Animal ID	FFPE block ID	Nested RT-PCR†		Tissue samples	ISH‡
				RdRp	Capsid		
GD	2016	GD-1	GD-1A	– (+)	– (+)	Spinal cord	+
						Brainstem	+
						Cerebellum	+
						Medulla oblongata	-
		GD-2	GD-2A	– (–)	– (–)	Lymph node	–
						Tonsil	–
						Myocardium	–
						Spleen	–
						Thymus	–
	2015	GD-11§	GD-11A	– (+)	– (+)	Brainstem	+
						Cerebellum	+
Tázlár	2011	TAZ-1	TAZ-1A	– (+)	– (+)	Hippocampus	–
						Brainstem	+
			TAZ-1B	– (+)	– (+)	Spinal cord	+
Balmazújváros	2014	BAM-1	BAM-1A	– (–)	– (–)	Spinal cord	–
			BAM-1B	– (+)	– (+)	Brainstem	+
						Cerebellum	+

*The screening RT-PCR primers are designed to either the RdRp or the capsid region of PoAstV-3. GD, index farm; ID, identification; ISH, in situ hybridization; PoAstV-3, porcine astrovirus type 3; RdRp: RNA-dependent RNA polymerase; RT-PCR, reverse transcription PCR; +, positive; –, negative. †Symbols indicate the results of the first screening PCRs; symbols in parentheses indicate the results of the second (nested) RT-PCR. The results of nested RT-PCR refer to a mixture of tissues embedded into the total of 7 paraffin blocks. ‡Indicates results for neuroinvasive PoAstV-3. §FFPE samples were the only specimens taken from this animal.

We also analyzed archived FFPE specimens from paraplegic pigs from earlier outbreaks of posterior paraplegia in Tázlár in 2011 and in Balmazújváros in 2014 (Table 2). The 3 swine farms are located in the central and eastern parts of Hungary, ≈100 km from each other, without known connection.

### Previous Laboratory Diagnostics

CNS homogenates from the index farm tested negative by PCR for the following pathogens (families in parentheses): porcine reproductive and respiratory syndrome virus (*Arteriviridae*); porcine circovirus 2 (*Circoviridae*); hemagglutinating encephalitis virus (*Coronaviridae*); and porcine parvovirus 1, 2, 4, and porcine bocavirus (*Parvoviridae*). Immunohistochemical detection of *Toxoplasma gondii* and West Nile virus and bacterial cultivation attempts from the CNS samples were also negative. Virus isolation attempts using brain homogenates of affected animals in swine kidney (PK-15) and Caucasian colon adenocarcinoma (Caco-2) cell lines were not successful (no cytopathic effects were visible). We detected no PoAsV type 3 (PoAstV-3) in the cell culture supernatants by nested RT-PCR with RNA-dependent RNA polymerase (RdRp) primer pairs.

### Total RNA Extraction and RT-PCR Screening

**Figure 1:**
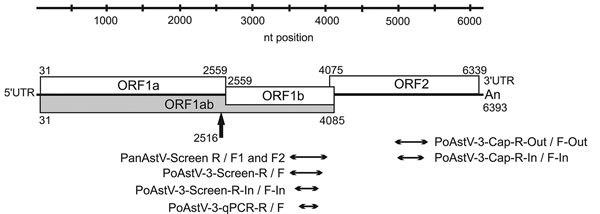
Genome map of the neuroinvasive PoAstV-3 strain NI-Brain/9-2016a/HUN (GenBank accession no. KY073229) from a symptomatic newly weaned pig from a farm in Hungary together with the location of RT-PCR products used for different astrovirus screening reactions and quantitative RT-PCR analyses. The black arrow indicates the possible localization of a ribosomal frame-shift during the synthesis of ORF1ab peptide. The first and last nucleotide positions of the ORFs are marked with numbers at the top and bottom of each box. ORF, open reading frame; PanAstV, panastrovirus; PoAstV-3, porcine astrovirus type 3; RT-PCR, reverse transcription PCR; UTR, untranslated region.

Treatment of FFPE samples included the deparaffination and rehydration steps, proteinase K digestion, and total RNA extraction. We used the same treatment protocols and the same reaction conditions and reagents used in the RT-PCR and nested RT-PCR reactions as are described previously, with minor modifications (*24**–**26*) (Technical Appendix). For the RT-PCR screening of CNS samples for the presence of pestiviruses (*Flaviviridae*) and swine picornaviruses (*Picornaviridae*), including teschovirus, enterovirus, sapelovirus, Seneca Valley virus, pasivirus, kobuvirus and encephalomyocarditis, we used virus-specific primer pairs as well as the outer and inner primer pairs targeting the RdRp or the capsid regions of PoAstV-3 (Figure 1; Technical Appendix Tables 1, 2).

### Absolute Quantification Using Quantitative RT-PCR

For the absolute quantification of viral RNA present in different tissue, urine, and fecal samples, we used the SYBR Green–based quantitative RT-PCR (RT-qPCR) method (Maxima SYBR Green qPCR Master Mix; Thermo Scientific, Waltham, MA, USA). For the generation of standard curve, we used 10-fold dilution series of purified and spectrophotometrically quantified RNA transcripts in the reactions. The RT-qPCR assays contained 3 technical repeats of all samples and standards. The slope of the standard curve was −3.4228 and the calculated PCR efficiency was 99.96%. The detailed protocol is provided in the online Technical Appendix.

### Long-range Amplification, 5′/3′ RACE-PCR, and Sanger Sequencing

For the complete genome (or complete 3′ open reading frame [ORF] 1b–ORF2–3′ untranslated region [UTR]) acquisitions of the PoAstVs, we used different long-range and 5′/3′ rapid amplification of cDNA ends RT-PCRs according to previously described protocols (*26**,**27*). We designed the sequence-specific primers used for the amplification of overlapping genome fragments based on the genome of PoAstV-3 strain US-MO123 (GenBank accession no. JX556691) and closely related sequences downloaded from the GenBank database (Technical Appendix Table 3). We sequenced PCR products directly with the BigDye Terminator v1.1 Cycle Sequencing Ready Reaction Kit (Applied Biosystems, Stafford, TX, USA) using the primer-walking method with an automated sequencer (ABI Prism 310 Genetic Analyzer; Applied Biosystems). We have submitted the nucleotide sequences of study astrovirus strains to GenBank under accession nos. KY073229–32.

**Table 3:** Amino acid differences between neuroinvasive PoAstV-3 strains from 3 symptomatic newly weaned pigs from a farm in Hungary and reference enteric PoAstV-3 strains detected from fecal samples*

Category	Genomic region									
	ORF1a	ORF1a	ORF1a	ORF1a	ORF1b	ORF1b	ORF2	ORF2	ORF2	ORF2
Amino acid position	1–400	1–400	401–844	401–844	1–508	1–508	1–415 (AD)	1–415 (AD)	416–754 (RID)	416–754 (RID)
PoAstV-3 type	Ni	Ent	Ni	Ent	Ni	Ent	Ni	Ent	Ni	Ent
Amino acid changes	M25	S/L	F408	L	N54	D	R29N	KT[I/A/V]	L439	H[P/V]
	Y41	F	I434	V	D106G	[A/E]D	S34	R	S453	D
	R117	K	S481	P	A181	S	R38	Y	F457	Y
	T120	S/L	S576	T/V	I206	V	V55	T	Y559	F
	T122	S/L	G608	N	R213	K	T57R	SK	A570[P]	N
	K151G	RC	N646	H	Y293	H/N	T61	A	N572[Y]	D
	L170	M	E679	D	E343	D			D581	N
	L179	M			K375	R			I601	V
	M185	L			I378	T			S617	N
	D208	E/N			N382	D			T628	S
	D202S[P/Q]–	NPTDG			I415	A/T			S678	T
	P217A	TT							I696	V
	T220[V/A]	IS								
	P224	H/R								
	I299	V								
	E332	D								
	V338	L/I								
	L346	F								
	I369	V								

*We identified 3 PoAstV-3 isolates: NI-Brain/9-2016a/HUN (GenBank accession no. KY073230); NI-Brain/173-2016a/HUN (accession no. KY073231); and NI-Brain/386-2015/HUN (accession no. KY073232). We compared these with enteric strains from GenBank (accession nos. JX556691, LC201595-7, and LC201599). AD, presumed particle assembly domain; Ent, enteric; Ni, neuroinvasive; ORF, open reading frame; PoAstV-3, porcine astrovirus type 3; RID, presumed receptor-interaction domain.

### Sequence and Phylogenetic Analyses

We aligned astrovirus sequences by using the MUSCLE web tool of EMBL-EBI (*28*) and performed pairwise nucleotide and amino acid identity calculations of the aligned sequences with GeneDoc version 2.7 (http://iubio.bio.indiana.edu/soft/molbio/ibmpc/genedoc-readme.html). We constructed phylogenetic trees of deduced amino acid sequence alignments by using MEGA version 6.06 software (*29*) and the neighbor-joining method with the Jones–Taylor–Thornton matrix-based model. Bootstrap values were set to 1,000 replicates, and only likelihood percentages of >50% were indicated.

### Histology and In Situ Hybridization

We performed chromogenic (with 3,3′-diaminobenzidine/DAB) in situ hybridization in FFPE slides (RNAscope 2.0, Brown Kit; Advanced Cell Diagnostics, Newark, CA, USA) according to the manufacturer’s instructions for viral RNA detection of Ni-PoAstV-3. We used 30 probe pairs generated at Advanced Cell Diagnostics designed to hybridize native viral Ni-PoAstV-3 RNA. Negative controls included Dap-B (dihydrodipicolinate reductase gene from *Escherichia coli* probe); an unrelated viral probe; and normal porcine brain region-matched sections. 

## Results

### Clinical Observations

There are ≈2,000 sows and their offspring in the investigated highly prolific index farm (GD). Episodes of neurologic disease of unknown etiology have persisted in the past 2 years. The syndrome affects an average of 30–40 weaned pigs monthly (1.5%–2% of total), although the number of monthly cases infrequently rose to ≈80 pigs (4%) in the autumn–winter seasons. The clinical signs of posterior leg weakness or paraplegia and pitching (stage 1); later paralysis of both legs and skin pain (stage 2); or loss of consciousness, paresis, and serious flaccid paralysis of muscles (stage 3) typically appear among weaned pigs 25–35 days old, 1 week after the weaning procedure (Video). We did not observe gastroenteric symptoms. All of the affected pigs in stage 3 of the disease were unable to eat or drink; they died due to exsiccosis (dehydration) or were euthanized. Signs persisted typically for 1 week before death or euthanasia. Postmortem examination results showed no signs of mechanical damage (fractures, abscesses, or hemivertebrae). Pigs are vaccinated against porcine circovirus 2, *Mycoplasma hyopneumoniae*, and *Actinobacillus pleuropneumoniae*. Preventive amoxicillin treatment of the piglets was done routinely at weaning. Due to the preventive measures in effect as of spring 2017, which included extensive decontamination of the piggeries and the physical separation of the newly weaned pigs from different litters, the number of encephalomyelitis cases among weaned pigs decreased with only 1–2 cases/month observed on the index farm.

**Video:**
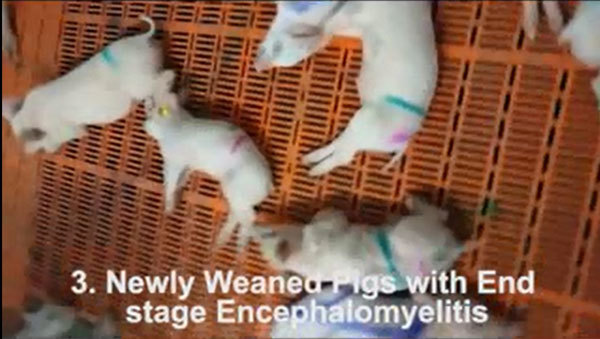
Stage 1: Weaned pig with posterior weakness. In milder cases and in early stage of the disease, lesions are confined to the lumbar part of the spinal cord, resulting in posterior weakness with motor incoordination, pitching, and fibrillary muscle twitching. The consciousness is intact and the behavior and interest of this pig toward his environment is normal, just imitating the so-called rooting behavior. Stage 2: Weaned pig with serious tetraplegia. The cervical, dorsal, and lumbar parts of the spinal cord are equally affected, producing serious movement disorder of both the front and hind legs. The consciousness is intact. Stage 3: Weaned pig with end-stage encephalomyelitis. The brainstem and cerebellum are most likely affected, resulting in loss of consciousness, paresis, and serious flaccid paralysis of muscles. The animals retain their sensory functions and react to pain.

The 2 additionally examined swine farms located in Tázlár and Balmazújváros each held approximately 500 sows and their offspring. Similar symptoms of staggering and paralysis appeared among pigs 3–5 weeks old in outbreaks in 2011 (Tázlár) and 2014 (Balmazújváros).

### Detection and Analysis of Astroviruses from CNS Samples of Affected Animals

In March 2016, we collected brain stem, spinal cord, nasal swab, and fecal samples from a newly weaned pig from index farm GD (GD-1, index animal) that showed signs of encephalomyelitis and posterior paraplegia (stage 1). The brain stem and spinal cord samples tested negative by RT-PCR for pestivirus (family *Flaviviridae*) and several swine-infecting picornaviruses (family *Picornaviridae*) (Technical Appendix Table 1). On the basis of the increasing evidence of the pathogenic role of neurotropic astroviruses among humans and farm animals (*5**,**6**,**14**,**17**,**30*) we investigated the presence of astrovirus using panastrovirus PCR primers (Technical Appendix Table 1) (*31*). The brain stem and spinal cord samples showed strong RT-PCR positivity. The panastrovirus PCR products were sequenced using panastrovirus PCR primers (Technical Appendix Table 1) and compared to each other and to the available astroviruses using blastn (https://blast.ncbi.nlm.nih.gov/Blast.cgi). The 397-nt sequences of brain stem and spinal cord were identical and showed 89% nt identity to PoAstV-3 isolate US-MO123 (GenBank accession no. JX556691) as the closest match (*32*).

We sequenced 2 samples from the index animal: the full-length genome of the neuroinvasive astrovirus strain NI-Brain/9-2016a/HUN (GenBank accession no. KY073229) from the brain stem sample and the complete capsid-encoding ORF2 from the spinal cord sample NI-SC/9–2016a/HUN (GenBank accession no. KY073230). The 6393-nt (without the poly[A] tail) complete genome showed the typical astrovirus genome organization with 3 putative ORFs, 2529 nt (ORF1a), 1527 nt (ORF1b), and 2265 nt (ORF2), flanked by short 5′ and 3′ UTRs (Figure 1). We identified the conserved proteolytic cleavage site (V_561_HQ↓TNT) of serine protease (ORF1a) and the conserved Y_358_GDD motif of the RdRp (ORF1b) (*33*). The nonstructural proteins of ORF1a (842 aa) and ORF1b (508 aa) and the capsid protein of ORF2 (754 aa) showed 93%, 95%, and 93% aa identity, respectively, to the corresponding genome parts of the closest known relative PoAstV-3 strain, US-MO123. All of the conserved genomic features of mamastroviruses were present in strain NI-Brain/9-2016a/HUN: the conserved C_1_CAAA pentamer at the 5′ end of the genome; the frame-shift heptamer motif (A_2511_AAAAAC) followed by a stem–loop structure at the 3′ end of ORF1a; the conserved sgRNA promoter sequence motif of U_4048_UUGGAGgGGaGGACCaAAN_8_AUGgC (variable nts are in lowercase, start codon of ORF2 is underlined) at the junction of ORF1b/ORF2; and the stem loop II-like motif (s2m) in the 3′ end of the genome between nt positions 6322 and 6353. The 3′ UTR of NI-Brain/9-2016a/HUN is 27 nt shorter and did not contain the short sequence repeat found at the 3′ end of strain US-MO123 (G_6381/6392_AUUUCUUUNA). Based on the high sequence identity and the similar genomic features, the NI-Brain/9-2016a/HUN strain most likely belongs to the PoAstV-3 genotype. The ORF2 of NI-Brain/9-2016a/HUN shares 99% nt/aa identity with the corresponding capsid gene of NI-SC/9-2016a/HUN from the spinal cord of the same animal, suggesting that the same virus was present in both regions of the CNS.

We detected Ni-PoAstV-3 using RT-PCR in all CNS samples collected from another 4 affected newly weaned pigs held in the index farm (Table 1). All of the samples from the asymptomatic control animals were Ni-PoAstV-3 negative.

We determined the complete genomes of 2 Ni-PoAstV-3 strains (NI-Brain/173-2016a/HUN, GenBank accession no. KY073231; and NI-Brain/386-2015/HUN, accession no. KY073232) that originated from 2 affected animals (GD-3 and GD-5) in stage 3 of the disease, chosen at different times (July 2016 and November 2015) of the outbreak (Table 1). These isolates showed 99.5%, 100%, and 98.7%–99.2% aa identities, respectively, to NI-Brain/9-2016a/HUN in the ORF1a, ORF1b, and ORF2 (capsid) regions.

**Figure 2:**
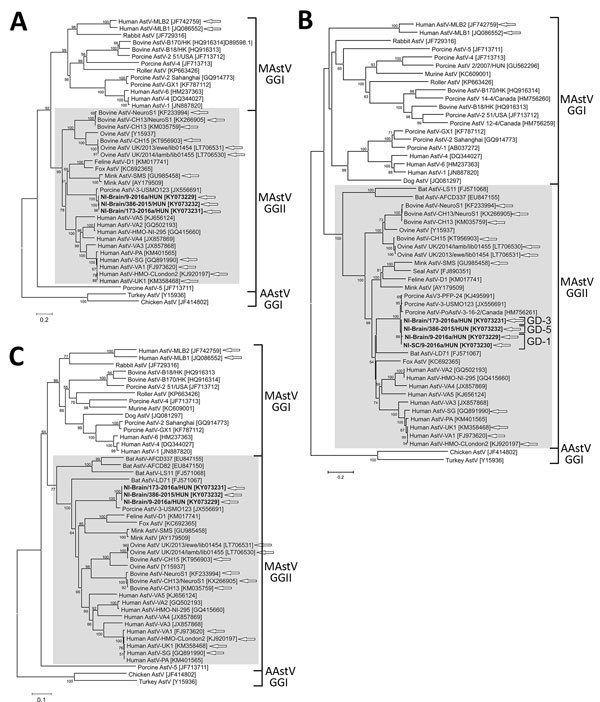
Phylogenetic analyses of the amino acid sequences of PoAstV-3 isolates (bold) from 3 symptomatic newly weaned pigs (GD-1, GD-3, and GD-5; see Table 1) from a farm in Hungary compared with reference isolates. A) ORF1a; B) ORF1b; C) ORF2. We included in the analysis available strains of the closest relatives (identified by blastx search [https://blast.ncbi.nlm.nih.gov/Blast.cgi]) of neuroinvasive PoAstV-3, all of the known porcine astroviruses with available complete coding sequences, all of the representative astrovirus strains with neurotrophic potential (white arrows), and some representative astrovirus sequences; GenBank accession numbers are in brackets. Gray boxes indicate the Virginia/Human-Mink-Ovine clade, which contains most of the neurotrophic astroviruses. Scale bars indicate amino acid substitutions per site. AAstV, avastrovirus; AstV, astrovirus; GG, genogroup; MAstV: mamastrovirus; ORF, open reading frame; PoAstV-3, porcine astrovirus type 3.

Most of the aa differences between the Ni-PoAstV-3 study strains and the other enteric PoAstV-3 strains are located in the N-terminal part of ORF1a and in the C-terminal part of ORF2 (Table 3). Phylogenetic analysis showed a close relationship between the identified Ni-PoAstV-3 sequences and the known PoAstV-3 strains located within the same larger clade containing most other mamastroviruses with known neurotropic potential (Figure 2).

### Detection of Ni-PoAstV-3 in Non-CNS Samples

We detected Ni-PoAstV-3 in multiple non-CNS samples from the respiratory system, lymphoid system, circulatory system, and salivary glands of affected animals (Table 1). We detected virus only in the second PCR round in 1 ileum sample and in 2 of the 3 analyzed fecal samples using nested RT-PCR (Table 1). Samples from internal organs (spleen and kidney) and urine samples tested negative by nested RT-PCR (Table 1).

**Figure 3:**
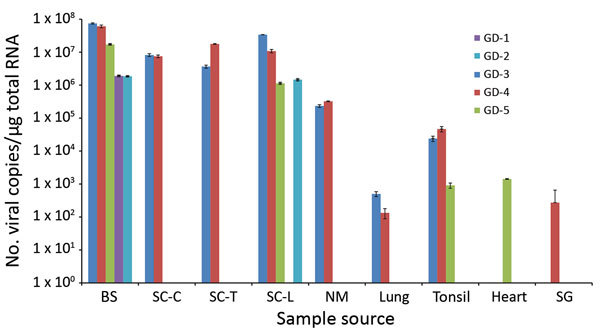
Logarithmic graph of the viral copy numbers of porcine astrovirus type 3 (PoAstV-3) in different organs determined by SYBR Green–based quantitative reverse transcription PCR (RT-qPCR) of samples from 5 symptomatic newly weaned pigs (GD-1–5; see Table 1) from a farm in Hungary. All the samples, which were positive for PoAstV-3 only by nested RT-PCR, were found negative by quantitative RT-PCR. BS, brain stem; CNS, central nervous system; NM, nasal mucosa; SC-C/T/L, cervical, thoracic, or lumbar spinal cord; SG, salivary gland.

We determined the copy number of Ni-PoAstV-3 using SYBR Green–based qPCR. All of the samples that showed nested RT-PCR positivity only in the second (nested) PCR round had negative test results by RT-qPCR, indicating low copy number (<100 copies/µg total RNA) of the virus in that tissue sample. The highest copy number was detected in the brain stem, followed by the spinal cord (Figure 3). Of note, we detected relatively high copy numbers in the tonsil and nasal mucosa samples (Figure 3). The serum of animal GD-3 contained 2.07 × 10^6^ virus copies/mL and of animal GD-4 1.64 × 10^3^ virus copies/mL.

To validate the general presence of Ni-PoAstV-3 in the respiratory system and the absence of the virus in the feces during the acute phase of the illness, we collected additional nasal and anal swab pairs from 5 affected pigs and 13 clinically healthy pigs of the same age (≈25–35 days) from the index farm. Four (80%) of the 5 nasal swab samples from affected animals tested positive but all of the anal swab samples tested negative using nested RT-PCR with primers targeting the RdRp region of Ni-PoAstV-3. The nasal and anal swab samples of the asymptomatic animals were all negative by nested RT-PCR. Because we collected varying amounts of samples by polyester-tipped swabs, we did not perform absolute quantification of Ni-PoAstV-3 by RT-qPCR.

### Detection of Ni-PoAstV-3 in Archived FFPE Samples

All but 1 archived FFPE samples from Tázlár and Balmazújváros were positive by nested RT-PCR for Ni-PoAstV-3 using 2 sets of primer pairs targeting the RdRp and capsid genes of Ni-PoAstV-3 (Table 2; Figure 1; Technical Appendix Table 2). The spinal cord FFPE sample from Balmazújváros had a negative result using both nested RT-PCR primer sets. The nested RT-PCR positive samples had positive results by ISH (data not shown).

### Histology and ISH

**Figure 4:**
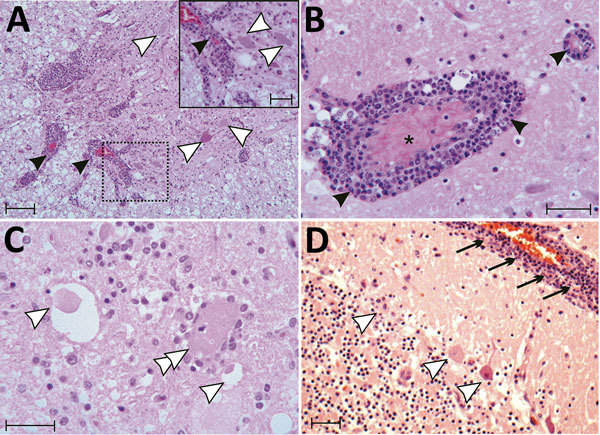
Tissue sections of cervical spinal cord (A), brain stem (B, C) and cerebellum (D) stained with hematoxylin and eosin from a symptomatic newly weaned pig from a farm in Hungary show the signs of stage 3 encephalomyelitis. Mononuclear perivascular cuffs with vasculitis (black arrowheads), neuronal necrosis (white arrowheads), neurophagia (white double arrowheads), multifocal microgliosis, and signs of meningitis (black arrows) are shown. Asterick (*) indicates blood vessel. Scale bars indicate 50 µm (panels A, D) or 20 µm (panel A inset; panels B, C).

**Figure 5:**
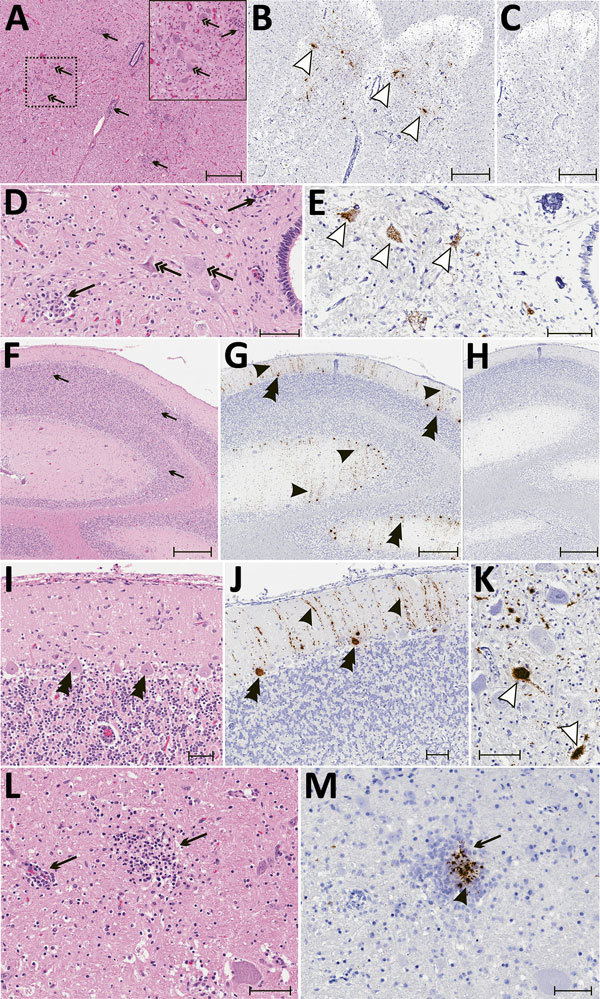
Results of histopathologic testing of central nervous system tissues from 2 symptomatic newly weaned pigs from a farm in Hungary. Sections of the cervical spinal cord (A–E), cerebellum (F–J), and cortex (L, M) from the index animal (GD-1) and the brain stem (K) from an additional affected stage 1 animal (GD-11). A, D, F, I, L) Hematoxylin and eosin stain. Gliosis (black arrows) is multifocal within the gray matter (panels A, D) and in the molecular layers (panels F, I, L and M). Neuronal degeneration and necrosis are evident by hypereosinophilia, angular degeneration, loss of neuronal detail, and vacuolation (double arrows in panels A, D). Some Purkinje neurons are slightly angular with mild vacuolation (double arrowheads in panel I). B, E, G, J, K, M) In situ hybridization of neuroinvasive porcine astrovirus. Hybridization of the neuroinvasive porcine astrovirus probe is restricted to neurons (white arrowheads in panels B, E, K) or limited to Purkinje neurons (double black arrowheads in panels G, J) with extension into dendritic processes that course through the molecular layer (black arrowheads in panels G, J). Hybridization of the neuroinvasive porcine astrovirus type 3 probe (black arrowhead in panel M) is present in the gliosis (black arrows in panels L, M). C, H) Using a control probe on a serial section, no hybridization is detectable. In situ hybridization. Scale bars indicate 500 µm (panels A–C, F–H) or 50 µm (panels D, E, I–M).

Histologically, shared CNS lesions among the animals examined were moderate to marked lymphohistiocytic cell perivascular cuffing with marked vasculitis and neuronal degeneration, necrosis, and neurophagia with multifocal microgliosis and satellitosis (Figure 4). The neuronal necrosis was especially evident in the dorsal and ventral horns of the cervical spinal cord gray matter, although it was also detected in neurons of the Purkinje layer (cerebellum), the medulla oblongata, cerebellar peduncles, and midbrain (Figure 5). Necrotic neurons were variously swollen and hypereosinophilic or shrunken with tinctorial changes including faded, amphophilic, or eosinophilic cytoplasm (Figure 5). Nuclei of affected neurons are pyknotic, karyorrhectic, or losing border definition within the cytoplasm. We performed ISH on 5 affected animals (Table 2). Ni-PoAstV-3 hybridization was predominantly restricted to neurons, including those with visible necrosis and, in the cerebellum in particular, some that were histologically unaffected, although some regions of gliosis (presumed inflammation after neuronal necrosis) also contained viral RNA (Figure 5, panel M). Hybridization was distinct, with punctate to diffuse cytoplasmic staining throughout the cytoplasm. The unique microarchitecture of the Purkinje layer of the cerebellum offered the clear demonstration that viral nucleic acid was present within dendritic processes coursing through the molecular layer (Figure 5, panels G, J). We found no pathologic lesions in other samples from kidneys, liver, gastrointestinal tract, or immune system (data not shown). The samples from the immune system were also negative by Ni-PoAstV-3 ISH (Table 2).

## Discussion

We detected astrovirus RNA in multiple tissues collected during 2015–2017 from newly weaned pigs with encephalomyelitis and posterior paraplegia of unknown origin, with the highest viral load detected in brain stem and spinal cord samples. We detected the same virus in archived brain and spinal cord FFPE samples from similarly affected animals from 2 additional swine herds collected in 2011 and 2014. These data indicate that a genetically similar, neurovirulent astrovirus is circulating in multiple swine farms since 2011 or earlier in Hungary.

According to the refined classification for the assessment of causation (*34*), the Ni-PoAstV-3 and the observed encephalitis and paraplegia are in a probable causal relationship (Level 2). Paraplegia associated with astrovirus neuroinfection is not unprecedented; minks had astrovirus-induced “shaking mink syndrome” and were reported paraplegic at the final stage of the disease (*12**,**35*).

Neurologic signs were observable mainly among newly weaned pigs (Video). The time of weaning, which involves nutritional (from milk to solid feed), social (mixing with different litters without the sow), and environmental (moving to a new pen) changes, is known to be the most stressful period in a pig’s lifetime and is associated with dysfunction of the immune system (*36*). Furthermore, the inadequate quantity and quality of colostrum intake of sucking piglets, and therefore the presumably low level of specific maternal antibodies due to highly prolific sows with large litters in the index farm, might also contribute to the emergence of the clinical disease. Decreased immune status was frequently present with extraintestinal dissemination of astroviruses in humans and in mice (*5**,**37**–**41*).

Our sequence analyses indicate that the identified astrovirus strains belong to the PoAstV-3 genotype, which clusters within the VA/HMO phylogenetic clade (Figure 2), as do most mammalian strains with known neurotropic potential (*6**,**14**,**19*). However, other canonical human astroviruses outside of the VA/HMO clade could also be associated with CNS disease (*41*). At the molecular level, the most conspicuous difference between the genomes of neuroinvasive virus and the enteric PoAstV-3 strain U.S.-MO123 is the 27 nt deletions found in the 3′ UTR of the CNS-associated astroviruses. The possible impact of this 3′ UTR deletion on viral tropism is unknown, although neuroinvasive bovine astroviruses also possess 3′ UTR architecture that differs from the diarrhea-associated astroviruses (*42*).

At the amino acid level, one of the most divergent regions between the neuroinvasive and other PoAstV-3 strains was found at the receptor-interaction domain of ORF2 (Table 3), which contains potential receptor binding sites (*43**,**44*). This finding could indicate an altered receptor spectrum and therefore altered tissue tropism of neuroinvasive and enteric PoAstV-3 strains.

PoAstV-3 strains were previously detected only from fecal samples of healthy or diarrheic piglets worldwide (*20**,**22**,**45*). We found that Ni-PoAstV-3 was either undetectable or detected only at low viral loads in the analyzed fecal samples, whereas the virus was generally detectable in the respiratory system of paraplegic pigs. This finding may indicate that CNS infection and replication occur later than enteric replication or that initial replication occurs extraintestinally (e.g., in the respiratory tract). Multiple types of astroviruses were recently identified from nasopharyngeal swabs or lung tissue samples from swine, bovines, and humans with respiratory symptoms including the neurotropic human VA1 strain from a patient with febrile acute respiratory disease (*9**–**11**,**23*), although neither the respiratory tropism nor the airborne transmission of astroviruses has been experimentally confirmed. Therefore, testing of only fecal samples from sick animals may result in underestimation of the incidence of astrovirus in pigs.

We measured the highest viral loads of Ni-PoAstV-3 in brain and spinal cord samples, similar to those found in diseased ovine and human patients with astrovirus-associated encephalitis (*5**,**14*). Ni-PoAstV-3 was also detectable in serum specimens and multiple organs of the respiratory, lymphoid, and cardiovascular systems of diseased swine. These results indicate that Ni-PoAstV-3 can result in viremia and disseminated infection involving the brain, spinal cord, and multiple organs during the acute phase of encephalomyelitis and posterior paraplegia. Astroviruses seem to play a role in a common and severe disease (encephalomyelitis and paralysis) in pigs.

The observable histopathologic changes, as well as the neuronal localizations of Ni-PoAstV-3 RNA in CNS samples of paraplegic pigs, are comparable to astrovirus-associated encephalitic cases of minks, humans, and cattle. Similar neuronal degeneration or necrosis with microgliosis in the brain or cerebellum, as well as inflammation of gray matter of the spinal cord, were previously described in cattle with astrovirus-associated nonsuppurative encephalitis (*6*,*35*,*46*,*47*), which suggests the general course of an astrovirus neuroinfection.

While some astroviruses are known to cause outbreaks of gastroenteritis, astrovirus-associated encephalitis cases have been reported only sporadically among humans, cattle, and sheep (*6**,**14**–**16**,**47*). The constant presence with recurrent increases of neurologic disease cases in swine farms indicates that natural neuroinvasive astrovirus infections may cause common, severe, persistent epidemics among domestic pigs and constitute an economically important agent threatening livestock and even humans, considering the possible zoonotic and recombinant potential of astroviruses (*48*).

Our results must be interpreted in the light of some potential limitations, which are currently true for other astrovirus-associated encephalitis studies: the absence of experimental evidence such as in vivo inoculation experiments, which could clarify the true causality between the astrovirus neuroinfection and the manifested CNS symptoms; and the roles of presumed respiratory replication and decreased immune state. Therefore, despite a growing body of scientific data regarding the presence of astroviruses in CNS in different animals, the direct association of astrovirus neuroinfection and encephalomyelitis should be treated with caution. Newly weaned pigs could potentially provide an in vivo animal model to study and clarify this association.

Technical Appendix Additional methods for testing of samples from newly weaned from a farm in Hungary for presence of neuroinvasive porcine astrovirus type 3.
