# Supplementary material for: Outbreaks of Neuroinvasive Astrovirus Associated with Encephalomyelitis, Weakness, and Paralysis among Weaned Pigs, Hungary
Source: Emerg Infect Dis. 2017 Dec;23(12):1982–93. doi: 10.3201/eid2312.170804 (PMC5708238; doi:10.3201/eid2312.170804)
Supplement: Technical Appendix — Additional methods for testing of samples from newly weaned from a farm in Hungary for presence of neuroinvasive porcine astrovirus type 3. [file 17-0804-Techapp-s1.pdf]

Article DOI: https://doi.org/10.3201/eid2312.170804

Outbreaks of Neuroinvasive Astrovirus Associated with Encephalomyelitis, Weakness, and Paralysis among Weaned Pigs, Hungary

Technical Appendix

Total RNA Extraction and RT-PCR Screening

We conducted pretreatment, including deparaffination and rehydration steps and Proteinase K digestion of FFPE samples, as described previously. (*1*,*2*).

For total RNA extraction, we used 50–100 mg of tissue samples, 400µL of Proteinase K–digested FFPE samples, and 150µL of urine, serum specimens, nasal swab samples, and fecal suspensions (35%–40% v/v, diluted in 0.1M PBS) prepared with TRI Reagent (Molecular Research Center, Cincinnati, OH, USA) according to the manufacturer's instructions. We ground the tissue samples manually to homogenize them using a Potter-Elvehjem pestle (Sigma-Aldrich Co., Munich, Germany).

We used the same reaction conditions and reagents in the RT and nested PCR reactions as were described previously, with minor modifications (*3*). Briefly, 1µg of total RNA was transcribed in a final volume of 25µL. We used the total amount of the RT product in the first PCR round in a final volume of 50µL. On RT-PCR negative samples, we performed a second PCR round with Ni-PoAstV-3 specific inner primer pairs for the specific and sensitive detection of small quantities of viral RNA. In the second (nested) PCR round, we used 1µL of the first PCR product as a template. The thermal program of the first PCR round contained 39 cycles, and the second, 35 cycles.

Absolute Quantification Using RT-qPCR

For the absolute quantification of viral RNA, we transcribed 1µg of total RNA, and for serum, urine, and fecal samples 2µL of total RNA using random hexamer (500ng/reaction) and

oligo dT (250ng/reaction) primers. For astrovirus quantification, we relied on primers of PoAsV3-qPCR-R/PoAsV3-qPCR-F designed for the highly conserved ORF1b using real-time PCR assay (Maxima SYBR Green qPCR Master Mix, Thermo Scientific, Waltham, MA, USA) (Figure 1 in main text; Technical Appendix Table 1). We followed the quantification steps of the qPCR reactions with a dissociation assay and ran both in a plate format on an ABI 7500 qPCR thermal cycler. For absolute quantification, a single PCR product generated by the PoAstV3-Screen-R and PoAstV3-Screen-F primers was cloned into a pTZ57R/T vector which contains a T7 promoter (InsTAclone PCR Cloning Kit, Thermo Scientific Waltham, MA, USA). We determined the orientation of the insert by sequencing. We linearized and transcribed the purified vectors with TranscriptAid T7 High Yield Transcription Kit (Thermo Scientific Waltham, MA, USA). After DNase-I treatment, we purified the transcribed RNA by phenol-chloroform extraction and ethanol precipitation. We used 10-fold dilution series of purified and spectrophotometrically quantified RNA transcripts in the RT reactions to generate standard curve.

Histology and *in situ* Hybridization

We designed 30 probe pairs generated at Advanced Cell Diagnostics (Newark, CA, USA) to hybridize to bps 4304–5246 of JX556692.1 within the PoAstV genome (ACD catalog No., V-PoAstV4-ORF2-C2) and to hybridize native viral Ni-PoAstV-3 RNA. As a positive control for nucleic acid integrity in the tissue sections, we used a probe that hybridizes to a conserved region of the highly expressed PPIB of *M. musculus* (Cat. #313919). Negative controls included Dap-B (dihydrodipicolinate reductase gene from *Escherichia coli* probe), an unrelated viral probe, and normal porcine brain region-matched sections. We conducted chromogenic *in situ* hybridization according to the manufacturer's instructions for nucleic acid detection (RNAscope 2.0 Brown Kit; Advanced Cell Diagnostics, Newark, CA, USA). We baked FFPE slides at 60°C for 1 hour, then deparaffinized with xylene twice, 10 min each time, followed by immersion in 100% ethanol twice before air-drying, then applied a hydrogen peroxide–blocking step, pretreatment 1, to the slides for 10 min at room temperature. We then boiled the slides in pretreatment solution 2 at 100°C for 15 min, followed by protease digestion for 30 min at 40°C to enhance target accessibility. We applied specific or control probes and then incubated the slides at 40°C for 2 h. We washed the slides twice with 1X wash buffer for 2 min at room temperature. We performed a series of 6 signal amplification steps according to manufacturer's instructions. We incubated

slides with 3,3'-Diaminobenzidine solution at room temperature for 8 min and quenched the reaction with dH_2O. We applied Gill's Hematoxylin for 2 min, rinsed slides in water and "bluing solution," and dehydrated them in 100% ethanol, 70% ethanol, and xylene before adding a coverslip.

References

1. Ma Z, Lui WO, Fire A, Dadras SS. Profiling and discovery of novel miRNAs from formalin-fixed, paraffin-embedded melanoma and nodal specimens. J Mol Diagn. 2009;11:420–9. PubMed http://dx.doi.org/10.2353/jmoldx.2009.090041

2. Ma Z. Total RNA extraction from formalin-fixed, paraffin-embedded (FFPE) blocks. Bio-protocol. 2012 [cited 2017 Sep 20]. 2012:2(7): e161 http://www.bio-protocol.org/e161

3. Boros Á, Pankovics P, Simmonds P, Reuter G. Novel positive-sense, single-stranded RNA (+ssRNA) virus with di-cistronic genome from intestinal content of freshwater carp (*Cyprinus carpio*). PLoS One. 2011;6:e29145. PubMed http://dx.doi.org/10.1371/journal.pone.0029145

4. Zell R, Krumbholz A, Henke A, Birch-Hirschfeld E, Stelzner A, Doherty M, et al. Detection of porcine enteroviruses by nRT-PCR: differentiation of CPE groups I-III with specific primer sets. J Virol Methods. 2000;88:205–18. PubMed http://dx.doi.org/10.1016/S0166-0934(00)00189-0

5. Boros Á, Pankovics P, Reuter G. Characterization of a novel porcine enterovirus in domestic pig in Hungary. Infect Genet Evol. 2011a;11:1096–102. PubMed http://dx.doi.org/10.1016/j.meegid.2011.04.003

6. Reuter G, Boldizsár A, Pankovics P. Complete nucleotide and amino acid sequences and genetic organization of porcine kobuvirus, a member of a new species in the genus *Kobuvirus*, family *Picornaviridae.* Arch Virol. 2009;154:101–8. PubMed http://dx.doi.org/10.1007/s00705-008-0288-2

7. Chu DKW, Poon LLM, Guan Y, Peiris JSM. Novel astroviruses in insectivorous bats. J Virol. 2008;82:9107–14. PubMed http://dx.doi.org/10.1128/JVI.00857-08

Technical Appendix Table1. Oligonucleotide primers used in study of porcine astrovirus in Hungary, 2017*

Target viruses	Oligonucleotide primer ID	Primer sequence (5′ → 3′)	Size of the PCR product (bp)	Reference
Pestivirus (*Flaviviridae*)	PoPestiV-Screen-R	TTT CCT CTG GCC CTG TTC TT	323	This study
Pestivirus (*Flaviviridae*)	PoPestiV-Screen-F	ACA GGR AAG AAY TGC CTG GT		This study
Teschovirus (*Picornaviridae*)	Teschovirus-5UTR-Rgen	CCA GCC GCG ACC CTG TCA GGC A	321	Zell et al., 2000†
Teschovirus (*Picornaviridae*)	Teschovirus-5UTR-Fgen	AGT TTT GGA TTA TCT TGT GCC C		Zell et al., 2000
Senecavirus and Cardiovirus (*Picornaviridae*)	SVV-Cardio-3D-screen-R	GTT GGC AGG MGT MAT CTT ATA	280	This study
Senecavirus and Cardiovirus (*Picornaviridae*)	SVV-Cardio-3D-screen-F	TCA GAT CYY TGG CTG TST CG		This study
Sapelovirus (*Picornaviridae*)	PSV-5UTR-F	CCC TGG GAC GAA AGA GCC TG	383	Zell et al., 2000
Sapelovirus (*Picornaviridae*)	PSV-5UTR-R	CCT TTA AGT AAG TAG TAA AGG G		Zell et al., 2000
Pasivirus (*Picornaviridae*)	SPaV-3Dscreen-R	CCA TGC ARA GCA AGC TCT AT	540	This study
Pasivirus (*Picornaviridae*)	SPaV-3Dscreen-F	GGT TAT GAT GGT TCT ATA CCA CG		This study
Enterovirus (*Picornaviridae*)	UnivEnt-5UTR-F	GTA CCY TTG TRC GCC TGT T	536	Boros et al., 2011‡
Enterovirus (*Picornaviridae*)	UnivEnt-5UTR-R	ATT GTC ACC ATA AGC AGC CA		Boros et al., 2011
Kobuvirus (*Picornaviridae*)	Univ-Kobu-F	TGG AYT ACA AGR TGT TTT GAT GC	216	Reuter et al., 2009§
Kobuvirus (*Picornaviridae*)	Univ-Kobu-R	ATG TTG TTR ATG ATG GTG TTG A		Reuter et al., 2009
Astrovirus (*Astroviridae*)	PanAstV-Screen-R	GGY TTK ACC CAC ATN CCR AA	443	Chu et al., 2008¶
Astrovirus (*Astroviridae*)	PanAstV-Screen-F1	GAR TTY GAT TGG RCK CGK TAY		Chu et al., 2008
Astrovirus (*Astroviridae*)	PanAstV-Screen-F2	GAR TTY GAT TGG RCK AGG TAY		Chu et al., 2008

*The numbers in the oligonucleotide primer IDs are the first (forward) or last (reverse) nucleotide positions of the target sites in the genome of Ni-PoAstV-3 NI-Brain/9–2016a/HUN. The PCR products were sequenced in both directions by primer-walking method using sequence-specific primers (data not shown)

†Zell R, Krumbholz A, Henke A, Birch-Hirschfeld E, Stelzner A, Doherty M, et al. Detection of porcine enteroviruses by nRT-PCR: differentiation of CPE groups I-III with specific primer sets. J Virol Methods. 2000;88:205–18. PubMed http://dx.doi.org/10.1016/S0166-0934(00)00189-0

‡Boros Á, Pankovics P, Reuter G. Characterization of a novel porcine enterovirus in domestic pig in Hungary. Infect Genet Evol. 2011a;11:1096–102. PubMed http://dx.doi.org/10.1016/j.meegid.2011.04.003

§Reuter G, Boldizsár A, Pankovics P. Complete nucleotide and amino acid sequences and genetic organization of porcine kobuvirus, a member of a new species in the genus Kobuvirus, family Picornaviridae. Arch Virol. 2009;154:101–8. PubMed http://dx.doi.org/10.1007/s00705-008-0288-2

¶Chu DKW, Poon LLM, Guan Y, Peiris JSM. Novel astroviruses in insectivorous bats. J Virol. 2008;82:9107–14. PubMed http://dx.doi.org/10.1128/JVI.00857-08

Technical Appendix Table 2. Porcine astrovirus 3 primers used in screening and quantification reactions*

Reaction type	Target genome region	Oligonucleotide primer ID	Primer sequence (5′ → 3′)	Size of the PCR product (bp)
Nested RT-PCR, first round	ORF1b - RdRP	PoAsV3-Screen-R	AAC CYT CTC CAC ATA ATT GGC	356
	ORF1b - RdRP	PoAsV3-Screen-F	GAA GTG TTT ATG CAC ATC AAG A	
Nested RT-PCR, second round	ORF1b - RdRP	PoAsV3-Screen-F-in	GCG AAG GAG TTT AGG ACG AA	190
	ORF1b - RdRP	PoAsV3-Screen-R-in	GGC GTA CTC AAA TGC TTG AA	
Nested RT-PCR, first round	ORF2 - capsid	PoAsV3-CAP-R-out	GGC CCA GTR TTA GGB GCA TT	256
	ORF2 - capsid	PoAsV3-CAP-F-out	ACC ATY TGG CAG ATA GTK GA	
Nested RT-PCR, second round	ORF2 - capsid	PoAsV3-CAP-R-in	CCA GTR TTA GGB GCA TTA ATC TG	250
	ORF2 - capsid	PoAsV3-CAP-F-in	CAT YTG GCA GAT AGT KGA TGA	
qPCR	ORF1b - RdRP	PoAsV3-qPCR-F	GGG TGA GGT AAC ATT GCA GA	100
	ORF1b - RdRP	PoAsV3-qPCR-R	GGC GTA CTC AAA TGC TTG AA	

*The numbers in the oligonucleotide primer IDs are the first (forward) or last (reverse) nucleotide positions of the target sites in the genome of Ni-PoAstV-3 NI-Brain/9–2016a/HUN. The PCR products were sequenced in both directions by primer-walking method using sequence-specific primers (data not shown).

Technical Appendix Table 3. Oligonucleotide primers used in genome acquisition reactions*

Reaction type	Oligonucleotide primer ID	Primer sequence (5′ → 3′)	Size of the PCR product (bp)
long range RT-PCR (3′ RACE)	PoAsV3–3403-F #	AAG CCT ACC AGT GGT ACT GCG A	2991
RT-PCR	PoAsV3–3420-R	AGT ACC ACT GGT AGG CTT CT	1755
RT-PCR	PoAsV3–1666-Fgen	ATG AGT GGK TCA CCW GTT AC	
RT-PCR	PoAsV3–1864-R	TGT CCT CAT GGC CAG AAG ACT	1579
RT-PCR	PoAsV3–286-Fgen	GGC GTC AAY GAR TGG GTT GA	
RT-PCR (5′RACE)	PoAsV3–5RACE-R2	GTT GCT GAG GAC GTA CAC GTT	469
RT-PCR (5′RACE)	PoAsV3–5RACE-R1	CCA ACA GAG AGC CAA TAA GTA A	
3′ and 5′ RACE	Anchored oligo dT-Adaptor	GAC CAC GCG TAT CGA TGT CGA C T(16) V	–
3′ and 5′ RACE	Adaptor	GAC CAC GCG TAT CGA TGT CGA C	

*The numbers in the oligonucleotide primer IDs are the first (forward) or last (reverse) nucleotide positions of the target sites in the genome of Ni-PoAstV-3 NI-Brain/9–2016a/HUN. # is used for the 3′ RACE RT-PCR reactions of NI-Brain/9–2016a/HUN, NI-SC/9–2016a/HUN, NI-Brain/173–2016a/HUN and NI-Brain/386–2015/HUN. The PCR products were sequenced in both directions by primer-walking method using sequence-specific primers (data not shown)
